# A study of red blood cell alloimmunization and autoimmunization among 200 multitransfused Egyptian β thalassemia patients

**DOI:** 10.1038/s41598-020-78333-y

**Published:** 2020-12-03

**Authors:** Amal El-Beshlawy, Alshymaa Ahmed Salama, Mohamed Roshdy El-Masry, Noha M. El Husseiny, Asmaa M. Abdelhameed

**Affiliations:** 1 Department of Pediatric Haematology, Faculty of Medicine, Cairo University, Cairo, Egypt; 2 Department of Blood Bank, El-Sahel Teaching Hospital, Cairo, Egypt; 3 Department of Internal Medicine and Clinical Haematology, Faculty of Medicine, Cairo University, Cairo, Egypt; 4 Armed Forces College of Medicine (AFCM), Cairo, Egypt

**Keywords:** Diseases, Medical research

## Abstract

The development of hemolytic erythrocyte alloantibodies and autoantibodies complicates transfusion therapy in thalassemia patients. These antibodies ultimately increase the need for blood and intensify transfusion complications. There is a scanty data on the frequency of RBC alloimmunization and autoimmunization in Egyptian β thalassemia patients as pretransfusion antibody screening is not routinely performed. We studied the frequency of alloimmunization and autoimmunization among 200 multiply transfused β thalassemia patients and investigated the factors that possibly affect antibody formation. Of the 200 patients in our study, 94 were males and 106 females, with the age range of 2–37 years. Alloantibodies were detected in 36 (18%) of the patients, while autoantibodies were detected in 33 (16.5%). The dominant alloantibodies were directed against Kell (33%) and Rh (24.4%) groups. Alloimmunization had a significant relationship with treatment duration and the frequency of transfusion (P = 0.007, 0.001, respectively). The presence of autoantibodies was significantly related to age (P = 0.001), total number of transfused units (P = 0.000) and splenectomy (P = 0.000). The high prevalence of alloimmunization in the study population disclosed the need for providing phenotypically matched cells for selective antigens especially for Kell and Rh subgroups to reduce risk of alloimmunization and increase the efficiency of blood transfusion.

## Introduction

Beta-thalassemia is among the most common autosomal recessive genetic disorders worldwide. Despite lack of reliable data for many regions of the world, globally, the estimated carrier rate is about 1.5%^1^, prevalence is ~ 288,000, and annual incidence of symptomatic individuals is 1 in 100,000^2^.

The burden of the disease is high in the Mediterranean basin and parts of Africa, throughout the Middle East, the Indian subcontinent, and Southeast Asia^3^. In Egypt, due to consanguineous marriage, the carrier rate is high, reported between 5.3 and ≥ 9%; with the annual incidence of the disease estimated to be 1000/1.5 million live births per year, making β thalassemia a significant health problem in Egypt^4^.

Although allogeneic hematopoietic stem cell transplantation (HSCT) is the only treatment with curative potential, few patients are considered for this treatment, due to lack of suitable donors, restrictions in eligibility requirements, and risk of transplant related mortality^5^. Appropriate and regular red cell transfusion remains the mainstay of treatment for severe forms of thalassemia; yet, its beneficial effects are limited by transfusion related problems such as viral infections, hemosiderosis and immunization against RBC antigens.

Alloimmunization to red cell antigens is a significant clinical challenge in the management of thalassemic patients who would otherwise benefit from transfusions. Alloantibodies may complicate the process of obtaining a suitable crossmatch compatible blood and decrease posttransfusion survival of RBCs. The concomitant presence of autoantibodies may further complicate these hazards.

The formation of alloantibodies is a common condition with variable prevalence ranging from 5.2 to 37% according to different studies^6–14^. The risk of alloantibody formation is influenced by genetic predisposition^15^, immune modulating conditions as infection^16^, and the number of transfused units^17^.

Extended matching would be a definitive solution for alloimmunization; however, the required costs and logistics bring up serious concerns in limited resources countries. A more practical and affordable approach is to apply partial better matching (PBM) for the most prevalent antigens.

Considering the adverse effects of alloimmunization and autoimmunization and challenges in their management, we conducted this study to evaluate the frequency of erythrocyte alloantibodies and autoantibodies, and specify the most frequent alloantibodies. The ultimate goal is to support the future consideration of applying partial better matching of the major culprit antigens responsible for alloimmunization in Egyptian multiply transfused β-thalassemia patients.

## Results

### Patient characteristics

Our study comprised a total of 200 patients including 94 males and 106 females. Their ages ranged from 2 to 37 years. The demographic and clinical characteristics of the study population are shown in Table 1. The ABO blood groups were in order of frequency: A (39.5%), B (22.5%), O (27%), and AB (11%). The RhD antigen was negative in 3.5% of the patients (Table 2).

**Table 1:** Demographic and clinical characteristics of the study population.

Variable	Number	Percent
**Gender**		
Male	94	47.0
Female	106	53.0
**Age**		
< 12 years	64	32.0
12–18 years	64	32.0
> 18 years	72	36.0
**Diagnosis**		
Thalassemia major	148	74.0
Thalassemia intermedia	52	26.0
**Age at start of transfusion**		
< 1 year	90	45.5
1–10 years	104	52.5
> 10 years	9	2.0
**Spleen state**		
Splenectomized	118	59.0
Non splenectomized	82	41.0

**Table 2:** ABO blood groups and RH type distribution.

Blood group	Number (percent)	Rh type	
		Positive	Negative
A	79 (39.5%)	76	3
B	45 (22.5%)	43	2
O	54 (27%)	53	1
AB	22 (11%)	21	1
Total	200 (100%)	193 (96.7%)	7 (3.5%)

### Screening and identification of alloantibodies

We detected alloantibodies in 36 (18%) patients, the number of alloantibodies per subject ranged from one to three, with the majority, 23 patients (64%), had three alloantibodies, while 10 patients (28%) had two alloantibodies and only 3 patients (8%) had one alloantibody.

A total number of 86 alloantibodies were identified, with 12 specificities, the majority (57%) of the antibodies were directed against K antigen and Rh system. Anti K was the most frequent (29; 33.7%), followed by antibodies against Rh antigens (20; 23.2%) [anti-E (7; 8.1%), anti-C (2; 2.3%), anti-Cw (11; 12.8%)]. Anti-Lea was detected in 11.6% (Table 3).

**Table 3:** The number and specificity of alloantibodies in 36 alloimmunized patients.

Type of antibody	Frequency	
	Number	Percent
**RH**		
Anti-C	2	2.33%
Anti-E	7	8.14%
Anti-Cʷ	11	12.79%
**Kell**		
Anti-K	29	33.72%
**Duffy**		
Anti-Fya	3	3.49%
**Kidd**		
Anti-Jkb	7	8.14%
**Lewis**		
Anti-Lea	10	11.63%
Anti-Leb	2	2.33%
**MNS**		
Anti-M	7	8.14%
Anti-N	1	1.16%
**Lutheran**		
Anti-Lua	2	2.33%
Anti-Lub	5	5.81%

### Alloimmunization rate in relation to patients’ characteristics

Table 4 summarizes the differences between the alloimmunized and non-alloimmunized patients in relation to the patient related factors deemed to be associated with RBC alloantibody formation. Overall, patients’ gender, diagnosis, age of starting transfusion, total number of blood units transfused, and spleen state did not show statistically significant differences between the alloimmunized and non alloimmunized patients.

**Table 4:** Frequency of alloantibodies in patient’s population in relation to demographic and clinical data.

Parameter	Alloantibodies		P value
	Present	Absent	
**Gender**			
Male	18 (19.1%)	76 (80.9%)	0.477
Female	18 (17.0%)	88 (83.0%)	
**Age**			
< 12 years	8 (12.6%)	56(87.5%)	0.064
12–18 years	8 (12.5%)	56(78.5%)	
> 18 years	20 (27.8%)	52 (72.2%)	
**Diagnosis**			
Thalassemia major	28 (18.9%)	120 (81.8%)	0.703
Thalassemia intermedia	8 (15.4%)	44 (84.6%)	
**Age of starting transfusion**			
< 1 years	17 (18.9%)	73 (81.1%)	0.873
	18 (17.3%)	86 (82.7%)	
> 10 years	1 (25.0%)	3 (75.0%)	
**Frequency of transfusion units/year**			
< 4/year	2 (15.4%)	11 (84.6%)	0.014
4–12/year	29 (17.7%)	135 (82.3%)	
> 12/year	5 (21.7%)	18 (78.3%)	
**Transfusion duration in months**			
< 160 m	12 (11.0%)	97 (89.0%)	0.001
≥ 160 m	24 (26.4%)	67 (73.6%)	
**Splenectomy**			
Splenectomised	25 (21.2%)	93 (78.8%)	0.346
Non splenectomised	11 (13.5%)	71 (86.5%)	

The prevalence of alloimmunization differed according to the frequency of transfusion per year: 21.7% of patients receiving > 12 units/year were alloimmunized compared to 17.6% of patients receiving from 4 to 12 units/year and 15.3% of patients receiving < 4 units/year (P = 0.014).

Alloimmunization was significantly higher in patients with transfusion duration ≥ 160 months 24/91 (26.4%) compared to 12/109 (11%) of patients with transfusion duration < 160 months (P = 0.001).

### Detection of autoantibodies

We detected RBC autoantibodies in 33 (16.5%) of 200 patients; 9 patients (27.2%) were in the alloimmunized group and 24 patients (72.8%) were in the non-alloimmunized group.

Table 5 summarizes the differences in frequency of autoantibodies according to patient related factors. Autoimmunization correlated with the patients’ age, autoantibodies could be identified in 19/72 (26.4%) patients > 18 years, compared to 12/64 (18.8%) patients from 12 to 18 years and 2/64 (3.1%) patients < 12 years (P = 0.001). Autoantibody development was also significantly associated with the duration (P = 0.001) and total number (P = 0.000) of transfusion. Splenectomized patients had significantly higher prevalence of autoimmunization (32/118; 27.1%), compared to (1/82; 1.2%) in non splenectomized patients (P = 0.000).

**Table 5:** Frequency of autoantibodies in patient’s population in relation to demographic and clinical data.

Parameter	Autoantibodies		P value
	Present	Absent	
**Gender**			
Male	12 (12.8%)	82 (87.2%)	**0.180**
Female	21 (19.8%)	85 (80.2%)	
**Age**			
< 12 years	2 (3.1%)	62 (96.9%)	**0.001**
12–18 years	12 (18.8%)	52 (81.3%)	
> 18 years	19 (26.4%)	53 (73.6%)	
**Diagnosis**			
Thalassemia major	26 (17.6%)	122 (82.4%)	**0.493**
Thalassemia intermedia	7 (13.5%)	45 (86.5%)	
**Age of start**			
< 1 years	15 (16.7%)	75 (83.3%)	**0.660**
	18 (17.3%)	86 (82.7%)	
> 10 years	0 (0%)	4 (2.4%)	
**Transfusion duration by months**			
< 160 m	9 (8.3%)	100 (91.7%)	**0.001**
160 m or more	24 (26.4%)	67 (73.6%)	
**Total number of transfusion**			
< 151	8 (7%)	107 (93%)	**0.000**
151 or more	25 (29.4%)	60 (70.6%)	
**Splenectomy**			
Yes	32 (27.1%)	86 (72.9%)	**0.000**
No	1 (1.2%)	81 (98.8%)	

## Discussion

Regular blood transfusion and iron chelation have dramatically improved life expectancy^18^ and quality of life for patients with β-thalassemia^19^. Alloimmunization is one of the most relevant post-transfusion complications as it is associated with transfusion delays, shortened in vivo survival of donor blood, and hemolytic transfusion reactions which can be fatal in some cases^20^. Recently RBC alloimmunization was identified as an independent predictor of HLA alloimmunization in HSCT with possible clinically significant adverse consequences^21^.

The existence of 35 blood group systems and more than 330 different blood group antigens realistically portray the massive diversity of red blood cell antigens specificities^22^ and could explain the formation of red cell antibodies.

Variable alloimmunization rates were reported in patients with thalassemia. In our study we demonstrated alloimmunization in 36 (18%) patients, which is in concordance with other Egyptian studies^23–25^ , Obeid et al.; however, found higher frequency of alloantibodies (42.5%) in the studied cohort in Alexandria province, Egypt^26^. El Danasoury and coworkers investigated the effect of limited donner exposure program (LDEP) on alloimmunization rate, and found alloantibodies in 3% of patients on LDEP compared to 21% of patients receiving transfusion from multiple donners^27^.

Alloimmunization has been linked to antigenic discrepancy between donner and recipients, Singer et al. reported a high alloimmunization rate (20.8%) among Asian patients and concluded that, among other factors, different racial background between white donors from United States and Asian recipients likely contributes to alloimmunization^28^. An even higher rate (30%) was demonstrated by Ameen and coworkers which was explained by heterogeneity of the population in Kuwait^29^. Recently, in January 2020, a study in Israel demonstrated 42.5% alloimmunization rate (17 out of 40 patients with transfusion dependent β thalassemia major)^30^.

The burden of RBC alloimmunization is likely underestimated as only a fraction of RBC alloantibodies formed are identified, as a result of peculiar RBC alloantibody induction and evanescence kinetics. Evanescence phenomenon has been found to affect different alloantibodies across antigenic specificities with considerable rates of disappearance^31^. Quantitation of antibody evanescence have largely focused on hospitalized patients with any diagnosis^32,33^ and sickle cell disease patients^34^.

It has been estimated that ≈ 30% of induced alloantibodies are regularly detected by antibody screen methods^31^, this fact underscores the need for complete transfusion records with multiple antibody workups which can be accessed across the health system.

Variable antibody specificity had been reported in different studies, attributable to variation in antigen frequency among different populations and the genetic effects on response to this antigenic stimulation. In numerous studies, the antigens of the K and Rh system were predominantly involved in stimulating production of alloantibodies, this predominance is expected considering the immunogenicity of these antigens^35^. In contrast, antibodies against Milternberger antigens were the most frequently reported in Chinese patients, due to its high prevalence in this population: 15% for Mia antigen and 6–7% for Mur antigen^36,37^.

We identified 12 antibodies specificities, the majority (92%) of patients had more than one alloantibody, with total number of 86 alloantibodies detected in our cohort. Anti K was the most frequent alloantibody detected (33.7%), followed by anti-Cw (12.8%) and anti-Lea (11.6%). Antibodies against Rh antigens collectively constituted 23.2% of alloantibodies detected [anti-E (8.1%), anti-C (2.3%), anti-Cw (12.8%)]. The predominance of Rh and Kell antibodies were previously demonstrated in Kuwaiti, Iranian, Omani, and Tunisian population^12,29,38,39^. It worth mentioning that the prevalence of K antigen was estimated to be 8.23% among Egyptian blood donors^40^.

Transfusion-related alloimmunization was found to depends mainly on the dose (the extent of transfusions and the absolute number of antigens per RBC) and immunogenicity of the antigen^41^ as the blood group antigen dose per RBC may vary from only a few to more than a million antigens per RBC^42^. However, the ability of developing red blood cell alloantibodies found to be restricted to a specific group of blood recipients, whose genetics and inflammation milieu favor the antigen presentation and augment the Th2 response^43^. Various patient-related factors may also influence the response to these antigens^16^; therefore, these variables should be explored in attempt to prevent this complex phenomenon.

Several studies reported a higher post-transfusion response rate in females, a finding that could not be confirmed in a meta-analysis, by Verduin et al., but was rather attributed to more allogenic exposure mainly because of pregnancy^44^. In our study, there was no significant difference in alloimmunization rate between males and females. This was also demonstrated by Thompson and colleagues when they assessed 697 patients with thalassemia on regular transfusion^9^.

In 1986, a study by Floss and coworkers demonstrated that infants do not produce alloantibodies even on exposure to many red and white cell antigens^45^. Later on, several studies showed significantly lower alloimmunization rate in patients who started transfusion therapy early^11,28^, this observation could be attributed to some form of acquired immune tolerance to red cell alloantigens^46^. Our study as well as others’ could not confirm this finding^47–50^; Pahuja et al. found immunization rate of 30% in patients who started transfusion before 1 year^7^.

Numerous studies showed higher alloimmunization rate in splenectomized than nonsplenectomized patients^27,28,51,52^. This difference proposed to be due to conformational changes in the RBC membrane which enhance immunomodulation leading to an increased risk of RBC allosensitization^28^; however, this finding may just reflect higher transfusion load. In the present study, no significant association was observed between splenectomy and the development of alloantibodies, similar to what had been reported in other studies^13,38,53^.

Autoantibodies formation in thalassemia patients had been previously reported in the literature^11,26,28,38,39,49,54^. The presence of RBC autoantibodies may interfere with pre-transfusion blood compatibility tests leading to difficulties in obtaining compatible blood and transfusion delay^39^. Clinically significant autoimmune hemolysis is a rare but potentially life threatening complication of autoimmunization^20^. We found autoantibodies in 16.5% of patients, autoantibody development was significantly associated with the patients’ age (P = 0.001). It was also related to duration (P = 0.001) and total number (P = 0.000) of transfusion. Splenectomized patients showed significantly higher prevalence of autoantibodies than non splenectomized patients (P = 0.000), this was previously reported by several researchers^11,28,55^ and supported by the finding of more erythrocyte-bound IgG in splenectomized thalassemia patient^56^.

Our study as well as others’ shed light into the need for strategies to prevent the production of allo and autoantibodies in order to maximize the effect of transfusion and avoid its complication.

Blumberg et al. in their work endorsed the hypothesis that leukoreduction of red cell transfusions alters its immunogenic effect in stimulating alloantibodies production against RBC antigens^57^. However; the role of leukodepletion in preventing erythrocyte alloimmunization and autoimmunization is not yet well confirmed, with various studies reveling contradictory results^14,28,57,58^.

Extended phenotype matching of transfusions considering antigens like Rh Cc Ee, K, Fy and Jk antigens had led to considerable reduction of the alloimmunization incidence in multiply transfused patients^28,38,59^.

Blood transfusion policy for thalassemia patients in Egypt require more efforts to control allo- and autoimmunization, in compliance with the international guidelines of blood transfusion in transfusion dependent thalassemia^60^, including provision of leukodereduced transfusion that is phenotypically matched for minor antigens with high frequency and proven significance. In addition, a regimen for follow up testing and documentation of alloantibodies in the transfusion records should be considered in attempt to mitigate delayed hemolytic transfusion reactions.

## Conclusion

Immunization to red blood antigens challenge the proper management of thalassemia patients. Alloimmunization and autoimmunization are common among multiply transfused thalassemia patients in Egypt. The most frequent alloantibodies were against Kell, Rh, Lutheran, and Lewis systems. Ideally, application of extended matched phenotyping can prevent alloimmunization; however, it’s financially and logistically demanding in limited resources health services, so adoption of a transfusion policy that provide phenotypically matched blood for the most frequent significant alloantigens (Rh and Kell) may offer a balanced cost-effective alternative to minimize red cell alloimmunization and autoimmunization among patients with thalassemia.

## Patients and methods

### Patients

This study was conducted in accordance with the code of conduct of research in Egypt and with the 1964 Helsinki Declaration and its later amendments. It was approved by Research Ethical Committee of Cairo university in October 2018. All participants or their parents and/or legal guardians (if under 18 years) gave informed consent to participate in the study.

This study included 200 multiply transfused β thalassemia patients, 148 (74%) thalassemia major and 52 (26%) thalassemia intermedia, registered in Abo-Elrish hospital and clinical hematology outpatient clinic, Kasr Alainy school of medicine, Cairo University. The study was conducted in 4 months’ duration from January to April 2019.

Clinical and transfusion records of all the patients were reviewed for demographic and clinical data including age, gender, age of initiating transfusion therapy, transfusion frequency, total number of blood units transfused, and status of splenectomy and these data were collected.

Patients were grouped according to age into three categories: < 12 years: 64 patients (32%), between 12 and 18 years: 64 patients (32%), and > 18 years: 72 patients (36%).

### Transfusion protocol

All patients were transfused with ABO and Rh (D) compatible, cross-matched, non leukoreduced blood (using CTD as preservative) with the aim to keep target Hb level 9–11.5 g/dl according to institutional transfusion policy.

## Methods

ABO typing was done by Colum agglutination technique (CAT) using Biovue system (Ortho Cassettes and Centrifuge).

Antibody screening was done using Ortho Surgiscreen reagent red blood cells which include most of the blood group of clinical significance. First 50 μL were added to each well, then 10 μL of (3–5%) cell suspension of reagent cells and 40 μL serum/plasma were added and the cassette was incubated for 10 min at 37 °C, then centrifuged for 5 min. The results are interpreted according to manufacturer’s instructions.

All positive samples were tested to identify the antibody specificity using Ortho RESOLVE panel A&B (11 cell panel in BioVue AHG Polyspecific Cassettes) based on Column Agglutination Technology as previously described by Delaflor-Weiss and Chizhevsky^61^. Patients’ serum was mixed with saline-suspended RBCs with the addition of low ionic strength saline and incubated at 37 °C degrees for 10 min.

Autoantibodies were detected by incubating patient’s own cell with patient’s plasma at 37 °C for 15 min and then centrifuging for 10 min on gel card containing poly specific antihuman globulin (anti IgG + C3d).

### Statistical methods

Data were statistically described in terms of frequencies (number of cases) and percentages and compared using Chi square (χ^2^) test. Exact test was used instead when the expected frequency is less than 5. p values less than 0.05 was considered statistically significant. All statistical calculations were done using computer program SPSS (Statistical Package for the Social Science; SPSS Inc., Chicago, IL, USA) release 15 for Microsoft Windows (2006).

### Compliance with ethical standards

All methods were done in accordance with the code of conduct of research in Egypt. This study was approved by the research ethical committee of Cairo university. All subjects or their parents and/or legal guardians (if under 18 years) gave written informed consent.

### Consent for publication

Verbal consents from all participants were obtained.

## Data Availability

All data are available.
